# Increased levels of RNA oxidation enhance the reversion frequency in aging pro-apoptotic yeast mutants

**DOI:** 10.1007/s10495-016-1319-1

**Published:** 2016-11-01

**Authors:** Mariarita Stirpe, Vanessa Palermo, Matteo Ferrari, Seweryn Mroczek, Joanna Kufel, Claudio Falcone, Cristina Mazzoni

**Affiliations:** 1 Department of Biology and Biotechnology “Charles Darwin”, Istituto Pasteur Italia-Fondazione Cenci Bolognetti, University “Sapienza” of Rome, Piazzale Aldo Moro 5, Rome, Italy; 2 Faculty of Biology, Institute of Genetics and Biotechnology, University of Warsaw, Pawinskiego 5a, 02-106 Warsaw, Poland

**Keywords:** Yeast, Apoptosis, Oxidative stress, RNA degradation, Genomic instability, Mutation frequency

## Abstract

Despite recent advances in understanding the complexity of RNA processes, regulation of the metabolism of oxidized cellular RNAs and the mechanisms through which oxidized ribonucleotides affect mRNA translation, and consequently cell viability, are not well characterized. We show here that the level of oxidized RNAs is markedly increased in a yeast decapping *Kllsm4Δ1* mutant, which accumulates mRNAs, ages much faster that the wild type strain and undergoes regulated-cell-death. We also found that in *Kllsm4Δ1* cells the mutation rate increases during chronological life span indicating that the capacity to handle oxidized RNAs in yeast declines with aging. Lowering intracellular ROS levels by antioxidants recovers the wild-type phenotype of mutant cells, including reduced amount of oxidized RNAs and lower mutation rate. Since mRNA oxidation was reported to occur in different neurodegenerative diseases, decapping-deficient cells may represent a useful tool for deciphering molecular mechanisms of cell response to such conditions, providing new insights into RNA modification-based pathogenesis.

## Introduction

DNA damage can lead to permanent sequence changes and many studies dealt with DNA damage checkpoint complexes and how cells handle damaged DNA. On the contrary, the possibility and the mechanisms of repairing damaged RNA and proteins has been considered unlikely to even exist for long time. The error rate of transcription is one mistake per 10^4^–10^5^ nucleotides about 10^5^ times higher than that of DNA replication. The lower fidelity of RNA synthesis and even lower fidelity of translation (~1 per 10^4^ amino acids) are tolerated because they cause no mutation. Damaged RNAs and proteins have thus been considered dispensable. Actually, considering the fact that the pool size of ribonucleotides is hundreds of times larger than that of 2′-deoxyribonucleotides [1], oxidized RNA precursors are more abundant than oxidized DNA precursors [2] and they can be incorporated into RNAs inducing translational errors and cell death [3, 4]. Recently, a growing number of studies suggests that RNA oxidation is an early event in a wide variety of neurological diseases, including Alzheimer’s disease (AD) and amyotrophic lateral sclerosis (ALS), Down syndrome and in the progressive loss of muscle mass and strength which occur during aging (sarcopenia) [5–8]. Uncovering on the consequences and cellular handling of the oxidized damaged RNA may provide significant insights into the pathogenesis of neurodegenerative diseases. Due to the complexity of studying these processes in mammalian model organisms, yeast, because of its easy handling and the high conservation of fundamental cellular pathways, could represent a convenient model to trace the basal root for determining the effects of RNA damage on cell physiology. We previously demonstrated that mutants in mRNA degradation, probably as a consequence of the stabilization and accumulation of certain mRNAs show premature aging, high level of intracellular ROS and apoptotic cell death features [9–12]. These mutants represent a convenient tool to study the effects of mRNA modifications during aging.

Moreover, since the discovery of yeast apoptosis [13] multiple yeast orthologs of crucial mammalian apoptotic proteins have been identified; conserved proteasomal, mitochondrial and epigenetically regulated cell death pathways have been outlined, and physiological death scenarios have been described [14–16], supporting the notion that the basic apoptotic machinery, including the one triggered by oxidative stress, is indeed present and functional also in unicellular organisms.

We also reported that cell death in decapping mutants, affected in early steps of mRNA degradation, is accompanied by elevated histone mRNA levels, persisting throughout the cell cycle, and is associated with defects in the S-phase progression [17]. The premature death of *Kllsm4Δ1* cells, expressing a truncated form of *KlLSM4*, the subunit of the decapping activator Lsm1-7 complex [9, 18], can be prevented by the reduction of histone mRNA levels following ectopic overexpression of the key histone transcriptional repressor Hir1p, as well by delaying the entry of cells in the S phase by low doses of hydroxyurea [17, 19]. Premature cell death in *lsm* mutants can also be prevented by the addition to the growth medium of antioxidant compounds, such as Acetyl-l-carnitine (ALC) or apple extracts [20, 21], suggesting that accumulation of ROS could play a central role in mediating aging and cell death in these mutants.

Here we present data concerning the accumulation of oxidized RNAs in the *Kllsm4Δ1* mutant and its effect on cellular aging and cell death.

## Materials and methods

### Strains and culture conditions

We used the *S. cerevisiae* strains MCY4/Kllsm4Δ1 (*Mat α, ade1-101, his3-Δ1, trp1-289, ura3, LEU2-GAL1-SDB23, pRS313*/*Kllsm4Δ1*), MCY4/313Kllsm4Δ1/LSM4 (*Mat α, ade1-101, his3-Δ1, trp1-289, ura3, LEU2-GAL1-SDB23, pRS313*/*Kllsm4Δ1 pFL44*/*LSM4*), CML39-11A (*Mat a, ade1-101, his3-Δ1, leu2, ura3, trp1-289*) [12, 19]. Cells were grown at 28 °C in YP (1 % yeast extract, 2 % peptone) supplemented with 2 % glucose (YPD), ethanol (YPE) or glycerol (YPGly) or in SD yeast nitrogen base without amino acids and auxotrophic requirement as needed. Solid media were supplemented with 2 % Bactoagar (Difco, Detroit, MI, USA).

### Northwestern analysis

Total RNA from yeast cells was isolated using a hot phenol procedure [22] and separated by electrophoresis on denaturing gel and transferred onto nitrocellulose filter. Northwestern was then carried out as previously described [23]. Anti-8OHG antibody (Ab) 15A3 (SantaCruz Biotechnology, CA, USA) were used.

### Polysome analysis

Polysomes were prepared from 100 ml of yeast culture grown to the late exponential phase (OD_600_ ~ 0.7) following the addition of cycloheximide to a final concentration of 100 µg/mL for 10 min at room temperature. Cells were washed twice with LB buffer (10 mM Tris-HCl, pH 7.5, 100 mM NaCl, 30 mM MgCl_2_, 100 μg/ml cyclohexymide, 200 μg/ml heparin, 0.2 μg/ml DEPC) and cell extract was prepared by vortexing cell suspension with 1 ml glass beads (425–600 μm, Sigma-Aldrich S.r.l. Milan, Italy) followed by centrifugation at 13.200 rpm for 10 min at 4 °C. RNA concentration was measured (NanoDropTM1000 Spectrophotometer; Thermo Fisher Scientific Inc. NYSE: TMO) and extract aliquots corresponding to 30 OD_260_ units in 400 µl were loaded onto a 7 and 47 % sucrose gradient prepared in buffer SG (50 mM Tris-HCl pH 7.5, 50 mM NaCl, 12 mM MgCl_2_, 1 mM DTT) using ACTA Prime chromatography system (GE Healthcare). Samples were centrifuged in the SW41 rotor (Beckman Instruments, Inc.) at 39,000 rpm for 90 min at 4 °C, gradients were fractionated by upward displacement with Fluorinert (3 M), using a gradient fractionator (Brendel, Alpha Biotech Ltd, Glasgow, UK) and ACTA Prime with fraction collector Frac-950.

In vivo protein labelling with ^35^S-labeled methionine (Hartmann Analytic GmbH, Germany) was carried out exactly as described [24].

### Mutation frequency analysis

For reversion frequency of Trp-phenotype and the occurrence of Canavanine resistant clones three independent cultures of the considered strains were cultivated at 28 °C in SD medium and auxotrophic requirement as needed. For each culture, three plates of selective medium lacking tryptophan or containing L-canavanine (3 µg/ml Sigma-Aldrich S.r.l. Milan, Italy) were inoculated and incubated at 28 °C for 3–5 days. The frequency of the Trp + reversion and of Canavanine resistant mutants was normalized to the number of viable cells in each experiment. Viability at day 1 was determined with the microcolonies test [25], while for the following days a standard colony forming units on YPD plates was employed. To measure RNA oxidation and Trp + reversion rate in the presence of antioxidant compounds 1 mM ALC and 26 mg/mL apple extracts [21] were added to the *Kllsm4Δ*1cell cultures. Total RNAs were extracted after 1 day of growth and analysed by Northwestern as described. Trp + reversion frequency was determined in the absence or in the presence of antioxidants.

### Statistical analysis

Figures 3, 4 and 5 show the mean of three independent experiments, Bar error indicates standard deviation; the number of stars (*) indicate the p value range: *p value <0.05, **p value <0.01, ***p value <0.001.

## Results

### Kllsm4Δ1 accumulates oxidized RNA during aging

**Fig. 1:**
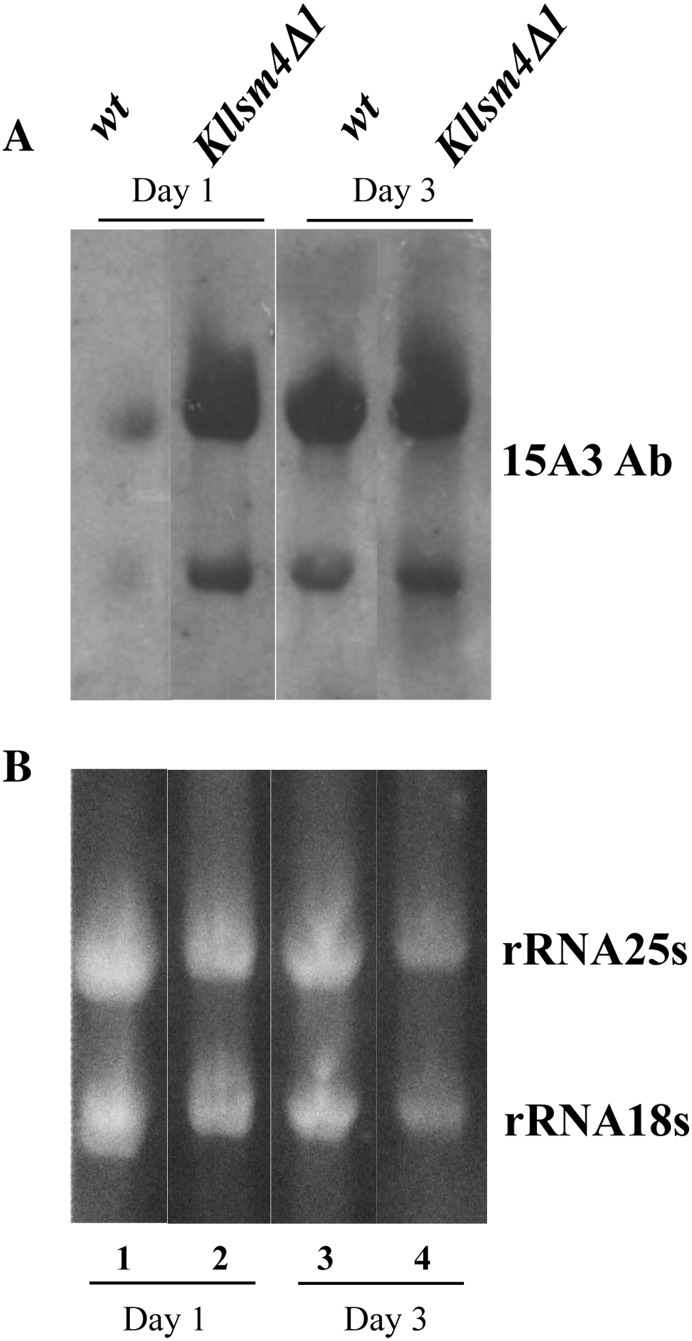
Oxidized RNAs accumulate during aging and in yeast decapping mutants. Total RNA was prepared from wt (CML39-11A) and *Kllsm4Δ1* cells after 1 day (*lane 1–2*) and 3 days (*lane 3–4*) of growth. RNAs were separated by gel electrophoresis and transferred to Opitran filter. **a** The presence of oxidized RNAs was revealed by the 15A3 antibody, which recognizes 8-oxo-7,8-dihydro- guanosine (8OHG). **b** Ethidium bromide staining was used to visualize ribosomal RNAs

**Fig. 2:**
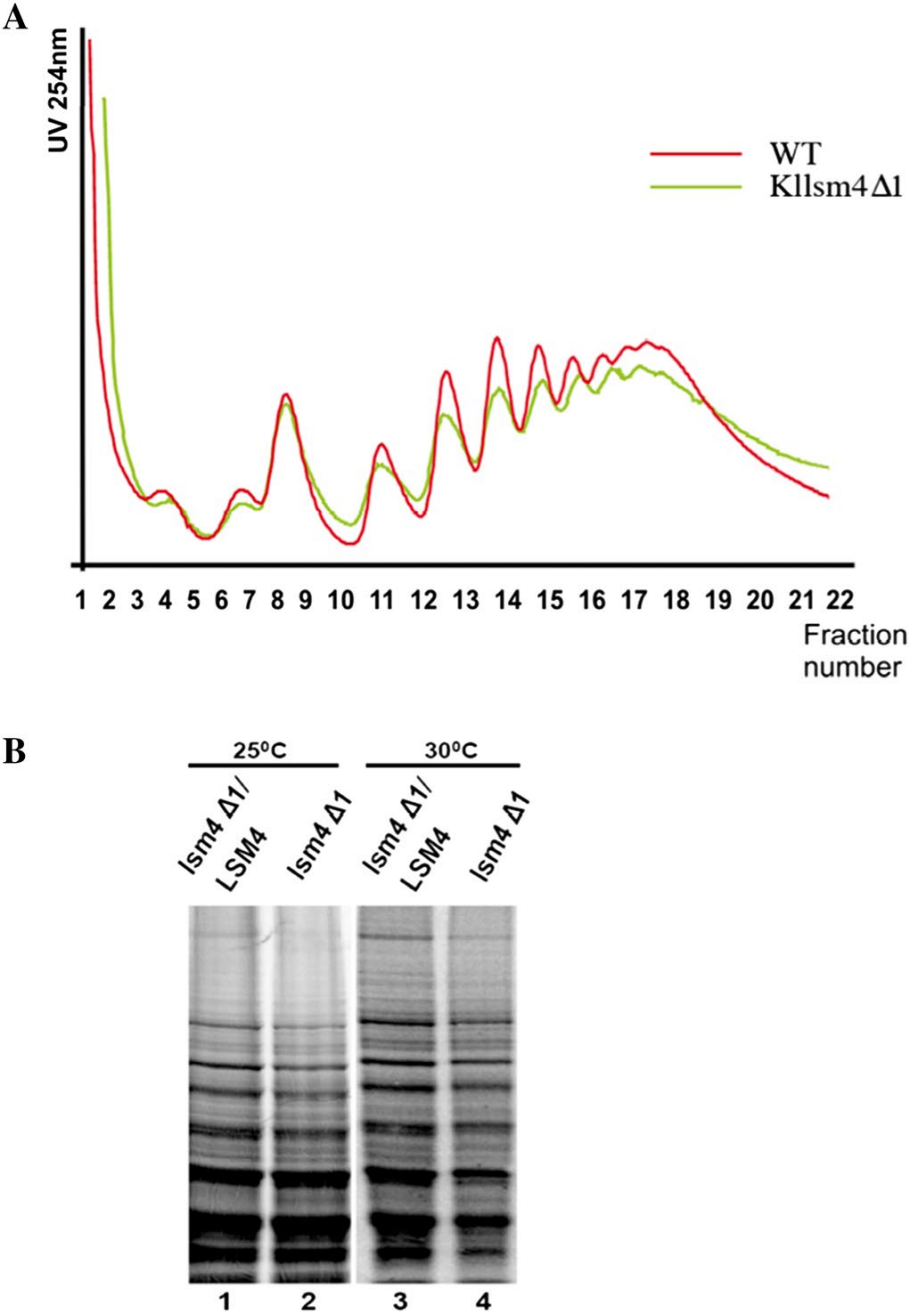
Polysome formation and translation capacity is not affected in the *Kllsm4∆1* mutant. **a** Polysome profiles of *Klsm4∆1* (*green line*) and *Kllsm4∆1*/*LSM4* (*red line*) strains grown in YPD medium at 30 °C. Equal amounts of cell extracts (30 A_260_ units) were fractionated by centrifugation in a 7–47 % sucrose gradient. Sedimentation is from left (*top*) to right (*bottom*). A_254_ was continuously measured during gradient harvesting. **b** Proteins were labeled with ^35^S-methionine *in vivo* in *Kllsm4∆1* and *Kllsm4∆1*/*LSM4* cells grown in SD-Met medium at 25 or 30 °C, separated in 12 % SDS-PAGE gel and visualized by autoradiography. (Color figure online)

We reported previously that yeast mRNA decapping mutants age prematurely and accumulate intracellular ROS [9–12]. The question arose whether or not the inefficient degradation of mRNA associated with the accumulation of ROS could have been related to the oxidation of mRNA. To answer this question, we isolated total RNAs from exponential and stationary phase cells and, by Northwestern analysis, we probed them with the antibody 15A3 which recognizes 8-oxo-7,8-dihydro-guanosine (8OHG), a marker of oxidative RNA damage. At the exponential growth phase, the amount of oxidized RNA was visibly higher in *Kllsm4Δ1* compared to the wild type strain CML39-11A, with ribosomal RNAs being the most evident (Fig. 1). Oxidized RNAs further increased in stationary phase cells, both in the wild type and in the *Kllsm4Δ1* mutant, indicating that RNA oxidation is linked to aging. We then verified the possibility that oxidized mRNAs might impair the correct formation of the translation machinery. To this end, we compared the polysome assembly in the *Kllsm4∆1*, the same strain expressing the wild type gene *LSM4 (Kllsm4Δ1*/*LSM4)* and we observed a very similar profile in both strains (Fig. 2a). Also the general translation capacity was not significantly compromised in the mutant, as assessed by metabolic pulse labelling of proteins with ^35^S-Methionine at 25 and 30 °C in *Kllsm4Δ1* and *Kllsm4Δ1*/*LSM4* cell extracts (Fig. 2b). However, in agreement with somehow reduced level of polysomes in the mutant, a slight decrease in the synthesis of larger polypeptides was observed. These experiments indicate that the amount of oxidized RNAs observed in *Kllsm4Δ1* cells does not significantly affect the efficiency of translation.

### Kllsm4Δ1 shows high reversion rate

**Fig. 3:**
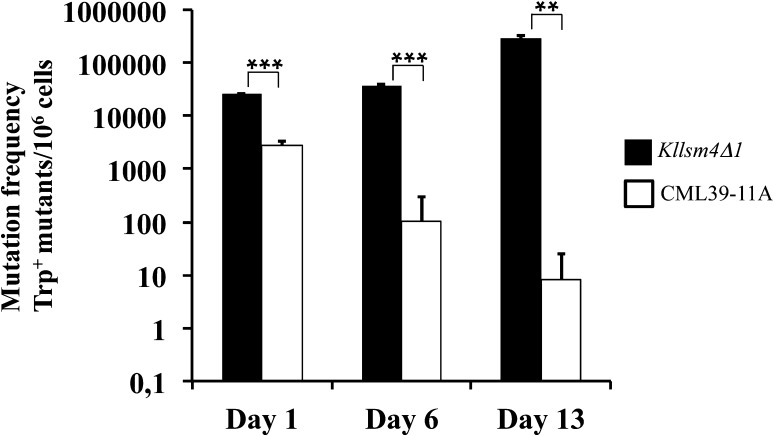
The *Kllsm4∆1* mutant shows higher mutation rate. Cells of *Kllsm4∆1* and wild type CML39-11A strains were plated on minimal medium after 1, 6 and 13 days of growth to determine the reversion frequency of the tryptophan minus (Trp^−^) phenotype. Plates were incubated at 28 °C for 4 days. Results are reported as the number of Trp^+^ revertants per 10^6^ living cells obtained from three independent experiments. **p value <0.01, ***p value <0.001

**Fig. 4:**
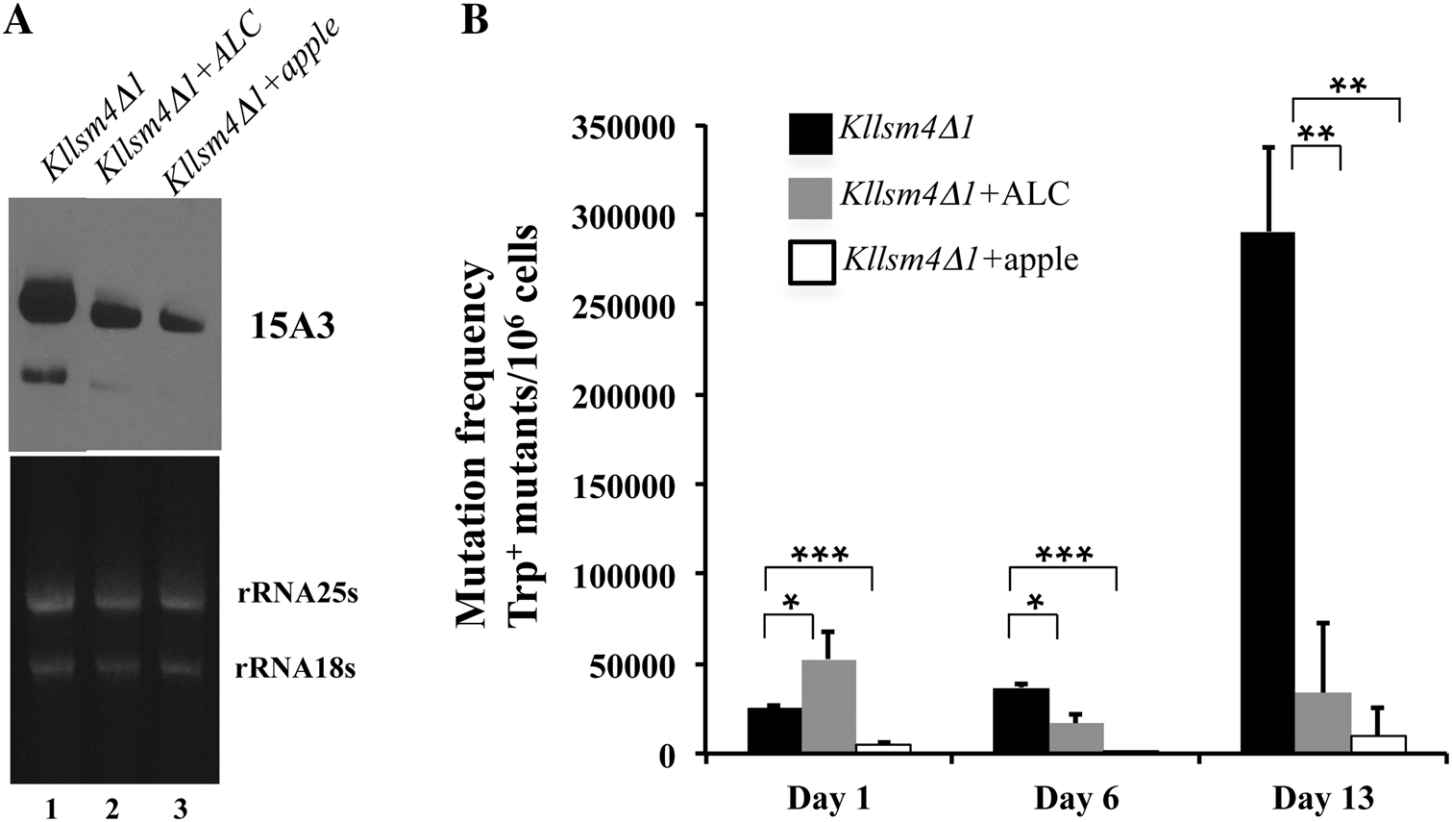
Antioxidant compounds can reduce RNA oxidation and the high reversion frequency of *Kllsm4Δ1*. **a** Total RNA was prepared from *Kllsm4Δ1* grown in the absence (*lane 1*) or in the presence of ALC and apple extract (*lanes 2* and *3*, respectively) and analysed as described in Fig. 1. **b** Cells of *Kllsm4∆1* grown in the presence of ALC and apple extract were plated on minimal medium after 1, 6 and 13 days of growth to determine the reversion frequency of the Trp^−^ phenotype. Plates were incubated at 28 °C for 4 days. Results are shown as the number of Trp^+^ revertants per 10^6^ living cells obtained from three independent experiments. *p value <0.05, **p value <0.01, ***p value <0.001. For a better comparison, results for the untreated *Kllsm4Δ1* cells, already shown in Fig. 3, were also included

To assess whether the presence of oxidized RNAs confers mutagenic effects, we compared the reversion frequency in *Kllsm4Δ1* and wild type strains of the *Trp1-289* allele, in which a C/T mutation in position 403 generates the amber TGA stop codon. Interestingly, we observed that the reversion frequency of the Trp^−^ phenotype increased in *Kllsm4Δ1* with aging and after 13 days of growth was about three orders of magnitude higher than in wild-type cells (Fig. 3). Sequence analysis of the *TRP1* gene amplified by PCR from 40 Trp^+^ clones did not reveal any additional mutations that could restore the open reading frame (data not shown). Similarly, no mutations were detected in the *SUP70* gene that codes for tRNA^Gln^_CUG_, whose mutations are known to suppress the UAG stop codon present in the *Trp1-289* allele [26] (data not shown). To verify a possible relationship between RNA oxidation and reversion frequency, we decreased the intracellular oxidative state, using our previous observations that antioxidant compounds, such as apple extracts and ALC, recover both apoptotic and premature aging phenotypes in *Kllsm4Δ1* [20, 21]. The analysis of RNA oxidation and the reversion frequency in the presence of these anti-oxidants demonstrated that both the extent of RNA oxidation and the number of Trp^+^ revertants were greatly reduced in the presence of apple extracts and ALC during cell growth (Fig. 4a, b). These results confirmed the hypothesis that the accumulation of oxidized RNAs contributes to the high reversion rate observed in the mutant. We then asked whether the difference in reversion frequency observed between the mutant and the wild type strains was due to a different mutation frequency, which is higher in aging cells compared to young cells [27].

**Fig. 5:**
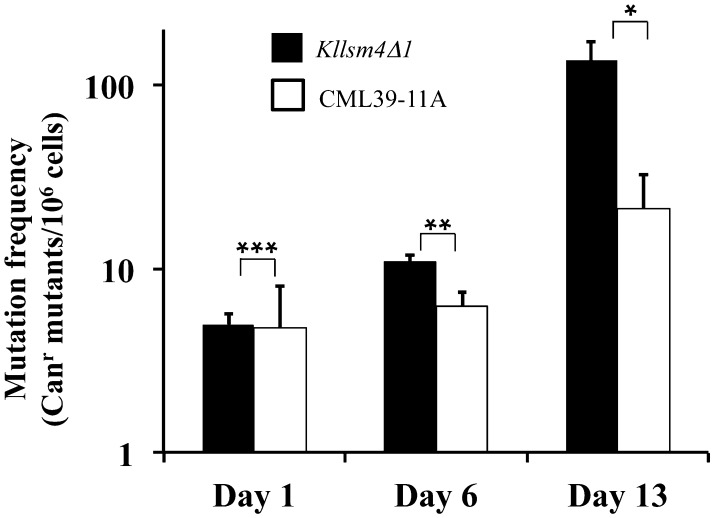
Mutation frequency of the *Kllsm4∆1* and the wild type CML39-11A strains. Cells were plated on minimal medium containing Canavanine sulfate after 1, 6 and 13 days of growth to determine the mutation frequency of the Can^r^ phenotype. Plates were incubated at 28 °C for 4 days. Results are reported as the number of Can^r^ mutants per 10^6^ living cells obtained from three independent experiments. *p value <0.05, **p value <0.01, ***p value <0.001

A common way to measure the mutation frequency in yeast is based on the canavanine resistance (Can^r^) phenotype originating from mutations in *CAN1*, the arginine permease gene involved in the uptake of arginine as well of its cytotoxic analogue canavanine [28]. To verify the occurrence of mutations, we sequenced the *CAN1* gene amplified from about 10 Can^r^ clones of each strain after 1 day of growth. Six out of ten clones showed the wild type *CAN1* sequence in the *Kllsm4Δ1* mutant, while base substitutions/insertions occurred in the remaining four clones, whereas the wild type base substitutions/insertions were observed in six out of seven clones in, with only one clone showing the wild type sequence (Table 1). These results indicate that the DNA mutation frequency in the *Kllsm4Δ1* mutant is similar to, or even lower, than that observed in the wild type. Sequencing of additional *Kllsm4Δ1*-derived Can^r^ clones after six days of growth revealed that 5 out of 11 clones had no sequence changes, a result very close to that observed after 1 day of growth. The remaining clones showed base substitution/insertion/deletion, three of which were already present on day 1, suggesting that their appearance occurred in the early stages of growth (Table 2). The observed mutation frequencies do not justify the abnormal occurrence of Trp^+^ revertants reported above, suggesting the involvement of other mechanisms enhancing the reversion frequency (Fig. 5).

**Table 1:** Sequence analysis of the *CAN1* gene from canavanine resistant *Kllsm4Δ1* and CML39-11A clones obtained from 1 day cultures

Strain	Clone	Mutation type			Position from ATG	Sequence
Kllsm4Δ1	1	Base substitution	G → T	Vali-Phe	703	CATCAAA**G**TTTT
		Insertion	G		708	TTTTA-**G-**GCCATTA
	2	Base substitution	G → A	Glu-Lys	424	TTGGGT**G**AAATG
	3	Base substitution	C → T	Glu-STOP	568	AGTTGGC**C**AAGT
	4	Base substitution	G → T	Glu-STOP	679	TGAATTC**G**AGTT
	5	No change				
	6	No change				
	7	No change				
	8	No change				
	9	No change				
	10	No change				
CML39-11A	1	Insertion	T		496	CATTT**T**GGTGCG
	2	Base substitution	G → T	Gly-Cys	352	AACGCC**G**GCCCA
	3	Base substitution	G → T	Glu-STOP	226	TGAAGAT**G**AAGG
	4	Base substitution	G → T	Glu-STOP	226	TGAAGAT**G**AAGG
	5	Base substitution	A → C	Leu-Phe	330	TGGTTT**A**TCCAC
	6	Base substitution	C → G	Ser-STOP	11	CAAATT**C**AAAAG
	7	No change				
	8	Partially sequenced				
	9	Partially sequenced				

**Table 2:** Sequence analysis of the *CAN1* gene from canavanine resistant *Kllsm4Δ1* clones obtained from 6 days cultures

Clone	Mutation type			Position from ATG	Sequence
1	Base substitution	G → T	Val-Phe	703	CATCAAA**G**TTTT
	Insertion	G		708	TTTTA-**G-**GCCATTA
2	Base substitution	G → A	Glu-Lys	424	TTGGGT**G**AAATG
3	Base substitution	G → A	Glu-Lys	424	TTGGGT**G**AAATG
4	Base substitution	G → A	Trp-STOP	612	GGCATG**G**ATTAG
5	Insertion	CA	Frameshift	579–580	CATTCA-**CA**-ATTT
6	Deletion	T	Frameshift	672	TTACGG**T**GAATT
7	No change				
8	No change				
9	No change				
10	No change				
11	No change				

## Discussion

Despite the evidence that oxidative damage to RNA is associated with numerous disease states, this phenomenon has received relatively little attention [29]. It has been reported that cells with deficient no-go decay pathway show increased increased levels of 8-oxoG mRNA, suggesting that mRNA surveillance mechanisms may have evolved to cope with damaged mRNAs [30]. RNA oxidation, concomitant with ribosomal stalling and production of truncated proteins, occurs very early in neurodegenerative diseases. Mutated proteins originating from mis-translation of oxidized mRNAs may undergo incorrect folding resulting in the formation of protein aggregates, which are the hallmarks of degenerative brain diseases, and induce apoptosis.

We previously reported a link between mRNA decapping defects and the onset of premature aging and apoptosis in yeast cells [11, 12]. Although the apoptotic phenotypes in decapping mutants are well-defined, the molecular mechanisms underlying this phenomenon are far from clear. In this paper we report that *Kllsm4Δ1* mutant cells accumulate oxidized RNAs, starting from the early stage of growth, leading to genome instability, as indicated by the exceptional high reversion frequency of the Trp^−^ phenotype. Nevertheless, this was not reflected in an increase in DNA mutation frequency, suggesting that the abnormally high reversion observed in the mutant might result from post-transcriptional/translational defects.

We hypothesize that the mechanism underlying the suppression of the auxotrophic mutations under selective pressure may be due to translational errors induced by oxidized mRNAs. Consistently, we found that reducing the intracellular ROS level by the addition of antioxidants can recover the wild type situation with respect to the amount of oxidized RNAs as well the mutation rate in the *Kllsm4Δ1* mutant. Although preliminary, these results open an interesting scenario that correlates RNA oxidation and the onset of cellular response mechanisms during cell aging. In this respect, yeast decapping mutants might represent useful tools for understanding the effects of RNA and protein damage on the cellular lifespan.
